# Flight route EDR estimation using MHE to fuse three-dimensional wind information in the QAR data analysis

**DOI:** 10.1371/journal.pone.0323147

**Published:** 2025-05-08

**Authors:** Hongping Wei, Haosen Li, Weiling Liu, Zibo Zhuang, Jun Chen

**Affiliations:** 1 Information Center, China Southern Airlines Company Limited, Guangzhou, China; 2 Institute of Aviation Meteorology, Civil Aviation University of China, Tianjin, China; 3 School of Safety Science and Engineering, Civil Aviation University of China, Tianjin, China; 4 School of Electronic information and automation, Civil Aviation University of China, Tianjin, China; University of Tabuk, SAUDI ARABIA

## Abstract

To address the problem that vertical wind data can provide information only on turbulence fluctuations in the vertical direction, meaning that the consideration of insufficiently compre-hensive turbulence information leads to low accuracy in eddy dissipation rate(EDR) estimation based on vertical wind (VWE), the paper proposes a flight route EDR estimation using MHE to fuse three-dimensional wind information in the Quick Access Recorder(QAR) data analysis. Quality control is performed on QAR data to eliminate irrelevant features for computing vertical wind and the two horizontal wind components. The corresponding formulas are then applied to obtain three-dimensional wind data information. Subsequently, a multi-head attention mechanism is employed to quantify the relationship between three-dimensional wind characteristics and the feature matrix (vertical acceleration). This process generates feature fusion weights, which are mapped onto the three-dimensional wind matrix and used to convert the three-dimensional wind data into one-dimensional wind data, thereby achieving the fusion of three-dimensional wind features. Finally, through maximum likelihood estimation(MLE) combine the new wind data in the frequency domain, the estimation of the flight route EDR is realized. Experimental results confirmed that the proposed estimation algorithm exhibits optimal performance compared to EDR estimations based on PCE and VWE, and the estimated values have smaller errors relative to the true EDR values. The proposed algorithm provides highly accurate EDR estimations for flight paths and demonstrates good practical applicability for assessing turbulence intensity along flight routes, thereby enhancing the safety of aircraft during flight.

## 1. Introduction

With the continuous warming of the global climate, the intensity and frequency of atmospheric turbulence are increasing [1,2]. Atmospheric turbulence can cause sudden bumpiness in aircraft flight and even lead to casualties in extreme cases [3,4]. Statistically, turbulence accounts for up to 65% of weather-related aviation accidents [5], which are the majority of flight accidents [3]. The objective and accurate identification of route turbulence is highly important for understanding and even predicting turbulence. Particularly, quick access recorder (QAR) is used to record real flight data captured by an aircraft’s sensors [6,7]. It can provide accurate turbulence information such as flight height, vertical acceleration and vacuum speed in real time, thereby providing the necessary data basis for accurate estimation of turbulence intensity [8,9].

The World Meteorological Organization uses the derived equivalent vertical gust velocity (DEVG) [10] and the eddy dissipation rate (EDR) [11] as indices for detecting turbulence on a route [12]. The DEVG has the advantages of few calculation parameters and being convenient to calculate, but as a gust load transfer factor, it is unable to eliminate the impact of the aircraft itself on turbulence assessment[13,14]. The EDR represents the disturbance state of the turbulence in the inertial subrange [15]. This index can eliminate the influence of aircraft and objectively identify atmospheric turbulence [16,17], which is highly important for avoiding turbulence-related risks and ensuring flight safety. The International Civil Aviation Organization uses the EDR as a detection index for atmospheric turbulence [18], and this index is widely used in conventional turbulence reports. With the increasing frequency of aviation turbulence events, the development of a more reliable EDR estimation method has become particularly important[19].

Currently, there are two main methods of EDR estimation algorithms [20]. The first is an EDR estimation algorithm based on vertical acceleration [21]. This algorithm obtains the response acceleration energy spectrum based on the vertical acceleration response function and estimates the EDR accordingly, but the vertical acceleration response function is usually determined through a linear fitting method, and its accuracy cannot be guaranteed [22]. The second is an EDR estimation algorithm based on vertical wind [23]. This algorithm estimates the EDR through maximum likelihood estimation of the vertical wind spectrum in the frequency domain. Because of its simple calculation, this algorithm has been widely used [24–27]. However, EDR estimation based on vertical wind may overlook the identification of certain types of turbulence occurring during actual flights, because various types of turbulence may be encountered on an actual route, such as convection induced turbulence [28], clear-sky turbulence [29,30], and others. In [31], Kim et al. input three-dimensional wind data into an EDR estimation algorithm and analysed different types of turbulence, it is evident that the EDR estimated based on vertical wind can identify convection-induced turbulence, but ignores clear-sky turbulence, while the EDR estimated based on horizontal wind has the opposite performance. These findings illustrates that the wind in different directions contains different turbulent fluctuation information [32,33]. In addition to vertical wind, horizontal wind can also interfere with aircraft balance, resulting in aviation turbulence [13,34]. Therefore, relying solely on EDR estimates based on vertical wind may underestimate or overlook turbulence. Through the fusion of three-dimensional wind features, more comprehensive information about turbulence can be obtained, thereby im-proving the accuracy of EDR estimation. In addressing the issue of feature fusion performance, many researchers have opted for the multi-head attention mechanism to fuse data features [35–43].

To tackle the issue that vertical wind data only reflects turbulence fluctuations in the vertical direction, resulting in an incomplete consideration of turbulence information, which in turn leads to lower accuracy in estimating the Eddy Dissipation Rate (EDR) based on vertical wind (VWE). The paper proposes a method for estimating flight route EDR in QAR data analysis by integrating three-dimensional wind information using MHE. First, quality control is applied to the QAR data to eliminate irrelevant features used in the calculation of vertical and horizontal wind components. Subsequently, the corresponding formulas are applied to obtain the three-dimensional wind data. Then, depending on the relationship between the structure and characteristics of QAR data, a multi-head attention mechanism is used to quantify the relationship between three-dimensional wind characteristics and feature matrix (vertical acceleration),This process generates feature fusion weights, which are mapped onto the three-dimensional wind matrix. These weights are then used to convert the three-dimensional wind data into one-dimensional wind data, thereby achieving the fusion of three-dimensional wind feature. Subsequently, through maximum likelihood estimation combine the new wind data in the frequency domain, the flight route EDR is finally estimated. The validity of the model is verified through comparison with the results of EDR estimation based on vertical wind and EDR estimation based on principal component estimation.

The contributions of this work are as follows: Firstly, the fusion of vertical wind and two horizontal wind components provides more comprehensive turbulence information. Subsequently, through maximum likelihood estimation in the frequency domain, the new one-dimensional fused wind data is integrated, significantly improving the accuracy of EDR estimation. Secondly, Based on Spearman analysis, the correlation between quality-controlled QAR data and EDR is examined, revealing that vertical acceleration has the strongest correlation with EDR. A feature matrix is constructed using vertical acceleration, followed by the use of a multi-head attention mechanism to quantify the relationship with the three-dimensional wind characteristics. This process generates feature fusion weights mapped to the three-dimensional wind matrix, enabling the conversion of three-dimensional wind data into one-dimensional fused wind data. Finally, the proposed algorithm provides high-precision EDR estimation for flight routes and demonstrates strong practical applicability in assessing the turbulence intensity along the route, thereby enhancing the safety of aircraft during flight.

## 2. Method

### 2.1. Multi-head attention mechanism

The multi-head attention mechanism consists of a multi-head attention layer and a fully connected layer. The multi-head attention layer divides the input data into multiple feature subspaces, allowing different attention weights to be assigned to data in different features, and selectively focusing on information from specific regions. This flexible weight allocation method demonstrates excellent fusion performance. [44–46].

The calculation process of the model can be regarded as the process of mapping the attention distribution between the query matrix Q and the key matrix K to the value matrix V to obtain the attention values. Specifically, the feature matrices $I^{q}$ , $I^{v}$ and $I^{k}$ are first projected into multiple feature subspaces to obtain Q, K and V, as shown in Equation (1).


$$\left\{ \begin{array}{c} Q\left[Q_{i},...Q_{h}\right]=\left[{W_{i}}^{q},...{W_{h}}^{q}\right]⬝I^{q}\\ K\left[K_{i},...K_{h}\right]=\left[{W_{i}}^{k},...{W_{h}}^{k}\right]⬝I^{k}\\ V\left[V_{i},...V_{h}\right]=\left[{W_{i}}^{v},...{W_{h}}^{v}\right]⬝I^{v} \end{array}\right.$$ (1)

$Q_{i}$, $K_{i}$, and $V_{i}$ are the split-head results for Q, K, and V, respectively; $W_{i}^{q}$, $W_{i}^{k}$and $W_{i}^{v}$ are one-to-one parameter matrices that can be learned. i=1,2⋯h represents the *i*th of h heads.

Second, the multiple independent single-head attention layers are used to process the data in the Q, K, and Vmatrices in the multiple feature subspaces in parallel. While this approach simplifies the calculation, it also allows the weights to be flexibly adjusted in accordance with the changes in local features to enhance the expressiveness of the model. Finally, the attention values (${head}_{i}$) output by each head are obtained.


$$head_{i}={\left(soft\max(\frac{Q_{i}K_{i}}{\sqrt{d_{k}}})\right)}^{T}*V_{i}$$ (2)

The Softmax(·) function is an activation function, that normalizes the third dimension of the attention score in the hidden layer output to a probability distribution of 0–1, thereby introducing a nonlinear transformation into the model to improve its nonlinear expression ability. The last two dimensions of the attention score obtained from the Softmax(·)function are transposed to correspond to the vectors in the $V_{i}$matrix. $d_{k}$ is a hyperparameter that prevents model overfitting, and its value is often consistent with the dimensionality d of the K matrix.

Finally, in the fully connected layer, the split-head results are merged by applying Equation (3) and Equation (4).


$$MulitiAttention(Q,K,V)=Concat(head_{i},...head_{h})⬝W$$ (3)

$$F=\frac{\sum_{j=1}^{d}MulitiAttention_{j}(Q,K,V)}{d}$$ (4)

**Fig 1:**
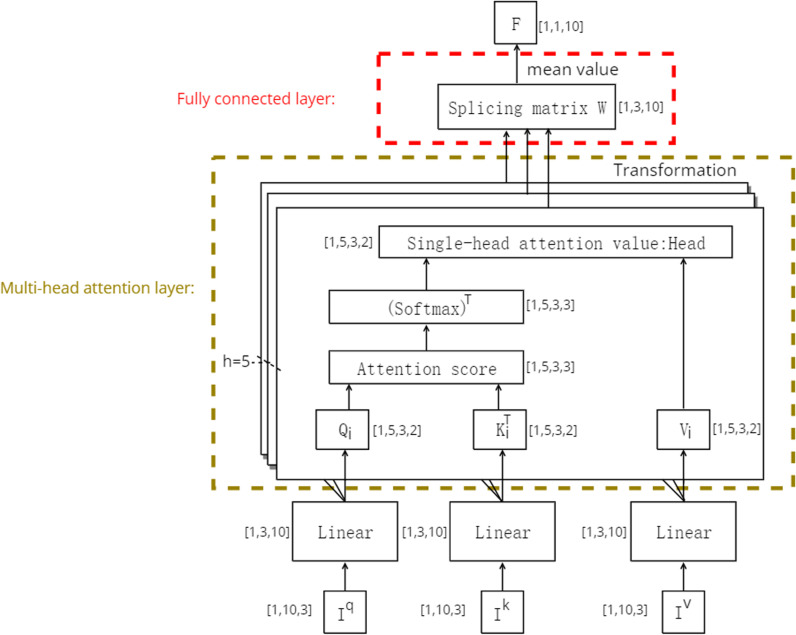
Internal structure diagram of the Multi-head mechanism.

Equation (3) uses the learnable splicing matrix W to combine the ${head}_{i}$ outputs into a set of multi-head attention values MulitiAttention. Equation (4) uses the mean method to transform MulitiAttention into one-dimensional output data. The internal structure of the model is shown in Fig 1, where the shape of the characteristic matrix is [1,3,10],and the number of split heads is 5.

### 2.2. Three-dimensional wind solution

The three-dimensional wind vector can be measured using data obtained from aircraft navigation systems (radio-assisted navigation systems, inertial platforms, magnetic compasses and GPS systems) and air speed systems (from Pitot hydrostatic systems and TAT probes). Specifically, the vertical wind is obtained from the vertical component of the inertial velocity, the vacuum velocity, the roll angle, the pitch angle and the left and right angles of attack [25]. The horizontal wind is determined based on the vacuum velocity, heading angle and ground velocity [33]. The calculation formulas are as follows:


$$w_{g}=-TAS*\left(\cos\theta \sin(AOA)\cos\varphi -\cos(AOA)\sin\theta \right)-IVV$$ (5)

$$U={\text{u}}_{g}-\left|TAS\right|*\sin\psi$$ (6)

$$V={\text{u}}_{g}-\left|TAS\right|*cos\psi$$ (7)

In these formulas, *U* and *V* are the two components of the horizontal wind decomposed along the true north direction, θ is the pitch angle, ∅ is the roll angle, and TAS is the vacuum velocity. *IVV* is the inertial vertical velocity of the aircraft, AOA is the body-axis angle of attack, $\left|{\vec{v}}_{a}\right|$ is the vacuum velocity, ψ is the clockwise heading angle relative to the true north direction, and $u_{g}$ and $v_{g}$ are the two components of the ground velocity decomposed along the true north direction.

## 3. Data

### 3.1. Data sources

In this paper, QAR data from a Boeing 737–800 aircraft collected during the period from January 2022 to June 2023 are used. The QAR data are composed of time series data of different frequencies, such as the left angle of attack (AOA1), right angle of attack (AOA2), pitch angle (Pitch_Angle) and other angle information, with a data update frequency of 8 Hz, and; the atmospheric static temperature (Static_Air_Temp), flight phase (FLIGHT_PHASE), vacuum speed (True_Air_Speed) and other parameters, with a data update frequency of 1 Hz; Moreover, the update frequency of the EDR is 0.1 Hz. To ensure the credibility, accuracy, and availability of QAR data, it is necessary to perform certain quality control procedures, specifically, identifying missing and invalid data, and deleting row data. In addition, the downsampling method is used to uniformly reduce the 8 Hz sampling frequency of the original data to 1 Hz to ensure the normal training and application of the model.

## 4. EDR estimation

### 4.1. Fusion of three-dimensional wind features

**Fig 2:**
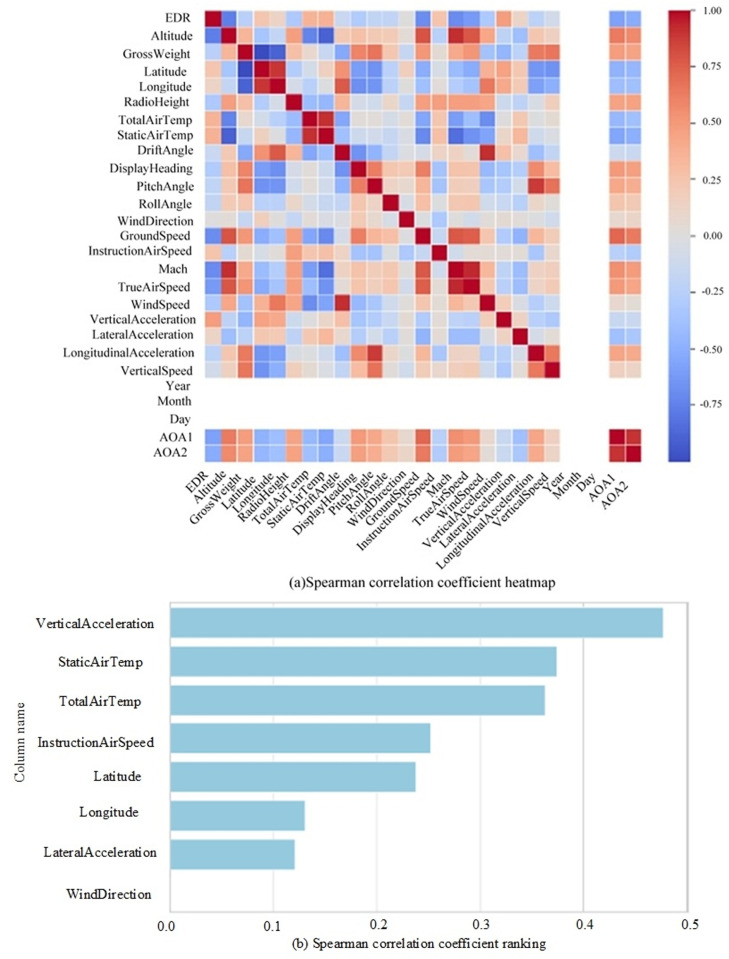
Heatmap and ranking of the correlations between various QAR data and the EDR. (a) Spearman correlation coefficient heatmap;(b) Spearman correlation coefficient ranking.

In the proposed method of EDR estimation based on a Multi-head attention mechanism (MHE), the Multi-head attention mechanism is used to quantify the relationship between the three-dimensional wind features and the feature matrix $I^{k}$, accordingly map feature fusion weights to a three-dimensional wind matrix, and then transform the three-dimensional wind data into one dimension to achieve feature fusion. As a key matrix for obtaining the weights, $I^{k}$ directly determines the quality of the fusion effect. In this paper, the Spearman analysis method is used to t analyse the correlations of the QAR data with EDR after quality control. The correlation analysis results are shown in Fig 2.

It can be seen from this figure that the correlations between the air temperature, atmospheric static temperature, vertical acceleration and the EDR are high. The correlation coefficients of the static air temperature and, total air temperature with the EDR are 0.37 and 0.36, respectively. The temperature gradient caused by temperature changes promotes air convection and, to some extent, the formation of turbulence when the vertical acceleration fluctuates violently while an aircraft is in the cruising phase, the aircraft encounters turbulence, and the greater the amplitude of the change is, the stronger the turbulence intensity [47,48]. Therefore, the vertical acceleration has a direct relationship with the EDR and the strongest correlation of 0.47.

**Fig 3:**
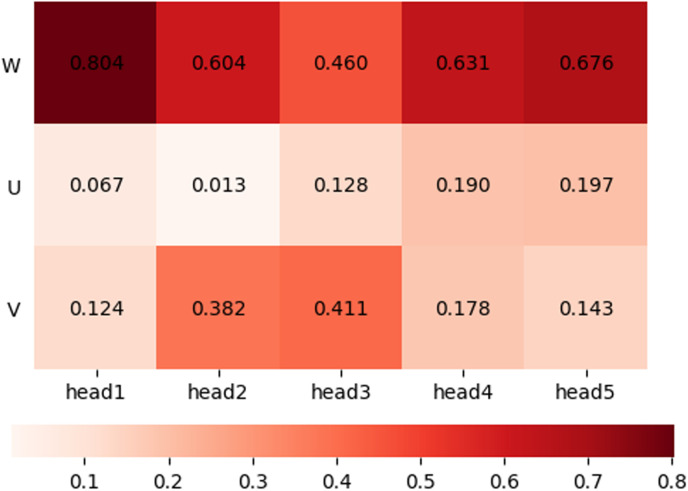
Feature fusion weights for three-dimensional wind features.

In this paper, the vertical acceleration, as the QAR variable with the strongest correlation with the EDR, is used to construct the feature matrix $I^{k}$. The number of heads of the model is determined to be 5 by the grid search method, and the time series length of the input feature matrix is 10 s. The three-dimensional wind data are projected into five feature subspaces; that is, the time series length in each feature subspace is 2 s. After five single-head attention layers, corresponding feature fusion weights are obtained for the three-dimensional wind features. By visualizing the weight distribution, the input features of the model and the importance of the feature sequence at different times can be analysed. Fig 3 shows a heatmap of the weights used in the process of feature fusion, which are updated in real time in accordance with the flight status.

The feature fusion weights for the vertical wind are between 0.460 and 0.804, accounting for the largest proportion; those for the horizontal wind feature U are between 0.067 and 0.197, and those for V are between 0.124 and 0.411. In general, these latter weights are slightly lower than the feature fusion weights for the vertical wind. This shows that compared with the traditional EDR estimation algorithm based on vertical wind, MHE additionally integrates the fluctuation characteristics of the horizontal wind to capture more comprehensive fluctuation characteristics. Through the split-head operation, more accurate control of the feature fusion weights can be achieved.

### 4.2. EDR estimation based on the Multi-head attention mechanism

Through the feature fusion weights, the three-dimensional wind features are fused to obtain new wind data. Then, to estimate the EDR, the traditional EDR estimation algorithm is used. The difference is that the input wind data are different. The EDR estimation algorithm applies a sliding window to the time series wind data and then performs MLE on the wind data in the window in the frequency domain to estimate the value of the EDR. The size of the sliding window is 10 [11,22], and the sliding step size is also 10. The estimation process is summarized in the following figure.

**Fig 4:**
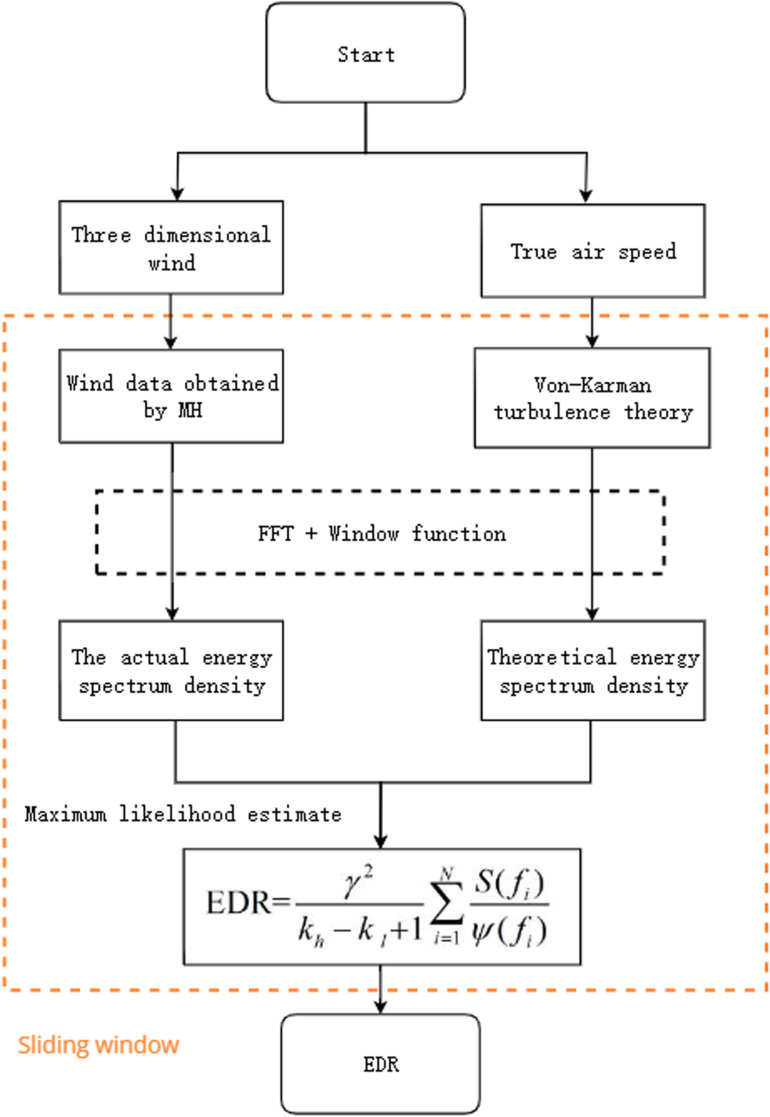
Flow chart of the EDR estimation algorithm.

In Fig 4, $S(f_{i})$ denotes the actual spectrum; γ is a correction factor related to the model; $k_{h}$ and ${\text{k}}_{l}$ are cut-off frequencies; and $\psi (f_{i})$ is the theoretical spectrum based on the Von Karman turbulence model, which is commonly applied as a theoretical model to estimate the EDR on flight route.

### 4.3. Model training

**Fig 5:**
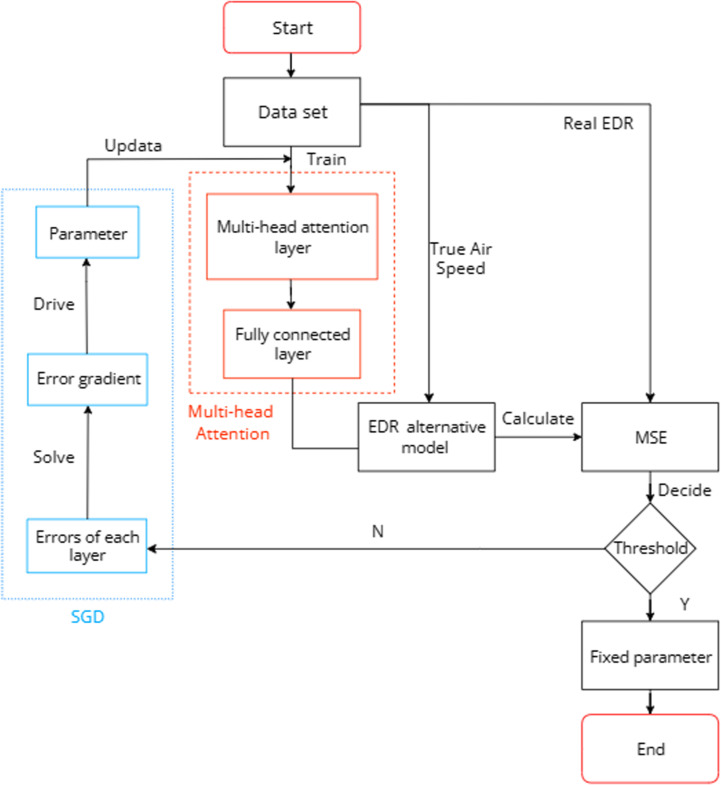
Training flow chart of the multi-head mechanism.

The fusion of the three-dimensional wind features is realized through the multi-head attention layer and the fully connected layer. The EDR value is output in accordance with the MLE method, and the model parameters are continuously updated by means of the stochastic gradient descent algorithm (SGD) along the direction of descent of the loss gradient to reduce the MSE loss value and improve the model performance. The specific training diagram is shown in Fig 5.

As stated above, the number of heads of the model is 5, and the time series length of the feature matrix and the correlation matrix is 10, consistent with the sliding window size in the EDR estimation algorithm. To improve the training efficiency, the number of samples used in each training epoch is set to 10, and the input tensor size is [4,10], which meets the input requirements of the network and optimizer. The dataset contains a total of 323,650 QAR data points and 32,365 EDR sample data points.

**Fig 6:**
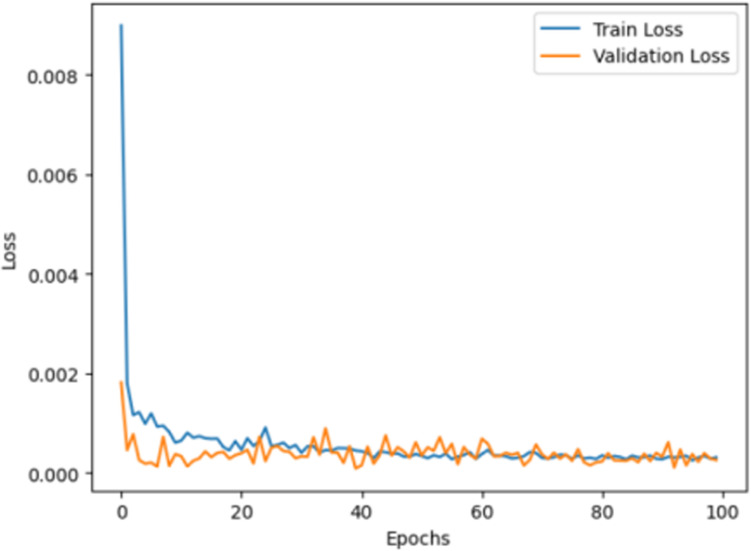
Loss curves of the multi-head mechanism.

It is divided into ten folds, six of which form the training sample set used to train the MHE model, while the remaining four are used as the verification sample set to evaluate the actual recognition performance of the model. The learning rate is set to 0.001, and the number of training epochs is set to 100. The loss curves on the training set and the validation set are shown in the following Fig 6.

As shown in Fig 6, in the early stage of training, the loss values on the training and validation sets continuously decrease and tend to stabilize after 40 epochs. The loss curves on the training set and validation set are in good agreement, the gap between the two is gradually reduced, and the overall training process is stable. This indicates that the model itself begins to converge, showing a good training effect.

## 5. Results

### 5.1. Experimental setup

The hardware used in the experimental setup of this study is equipped with a 12th Gen Intel(R) Core(TM) i5-12500H 2.50 GHz processor running at 2.50 GHz, and 16.0 GB of RAM. The programming language utilized is Python (version 3.9), and the operating system is 64-bit.

### 5.2. Analysis of the wind power spectral density

**Fig 7:**
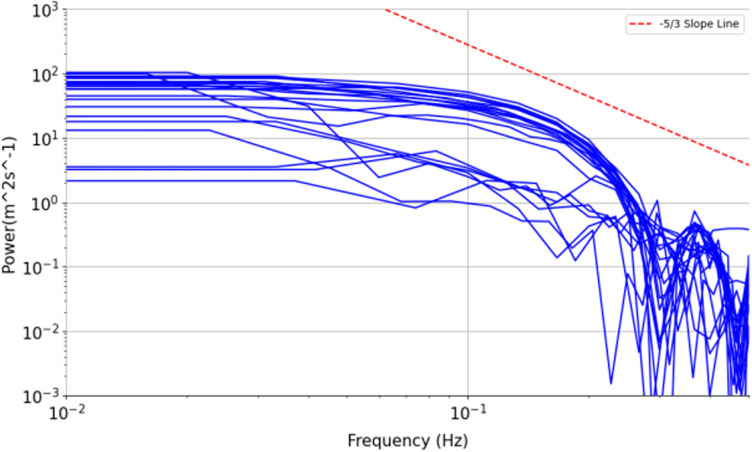
Power spectrum diagram.

The Von Karman turbulence model can describe turbulence within the inertial subrange and at larger scales beyond the inertial subrange and is commonly applied as a theoretical model to estimate the EDR on route [22,49].To ensure that the Von Karman turbulence model is applicable to the wind data output by the multi-head attention mechanism, a power spectral density diagram of the wind data is plotted in a logarithmic coordinate system with frequency on the abscissa and power on the ordinate, as shown in the following figure. Fig 7 shows the power spectral density curves of 25 wind data series with a time series length of 40 s after feature fusion.

As shown in Fig 7, for frequencies between the orders of 10 to the powers of −1 and −0.3 power of 10, the overall slopes of the power spectral density curves are is consistent with a reference the slope reference line of $-\frac{5}{3}$, which conforms to the assumption of isotropic turbulence assumption in the inertial subrange [50]. Therefore, the EDR can be estimated by means of the maximum likelihood estimation method in the frequency domain and the Von Karman turbulence model [22].

### 5.3. Comparative analysis of wind

In order to more intuitively reflect the fusion effect of the multi-head attention mechanism, two segments of mild turbulence data are randomly selected for wind speed comparison, and the relevant parameters are shown in the figure. The specific turbulence level, duration, and vertical overload peak data are shown in the Table 1. In addition, this paper considers the pursuit of stability of airliners. It is assumed that the turbulence shown by vertical overload is caused by the chaotic motion of the wind. In the following analysis, the influence of the pilot ‘s maneuverability is not considered.

**Table 1:** Introduction of turbulence field related content.

Turbulent field	Duration	Degree	Maximum/minimum vertical overload (g)
TF1	3340s-3686s	Weak	+0.972g/ + 1.104g
TF2	19740s-3567s	Weak	+0.926g/ + 1.135g

(1) TF1: On July 10, 2023, a Boeing 737–800 flying from Jinan Yaoqiang International Airport to Harbin Taiping International Airport encountered mild turbulence lasting one minute at 31,098 feet (9477.9 m). The variations in the wind and vertical acceleration during this period are shown in the following figure.

**Fig 8:**
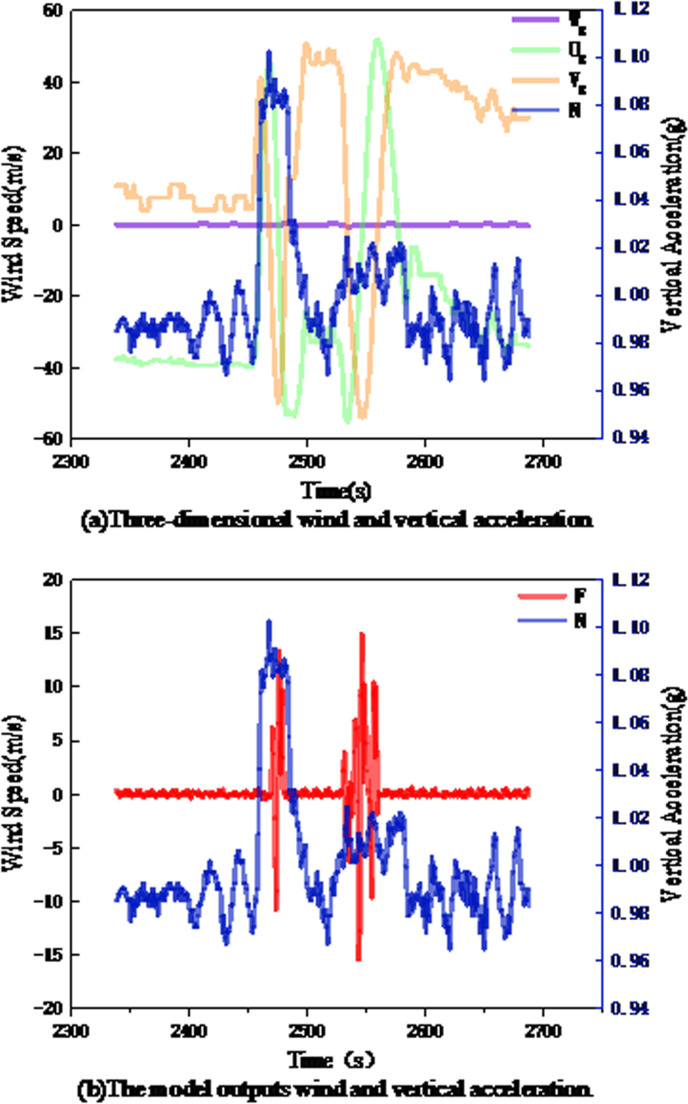
Wind speed and vertical acceleration of TF1 segment. (a)Three-dimensional wind and vertical acceleration; (b)The model outputs wind and vertical acceleration.

As shown in Fig 8(a), the change in the vertical wind W was relatively stable, and the fluctuation was basically 0, inconsistent with the change in the vertical acceleration, while the horizontal wind components (U and V) varied between 57 m/s and 50 m/s. These intense fluctuations in the horizontal winds are the cause of flight turbulence. As shown in Fig 8(b), the wind data (F) output by the multi-head mechanism integrates the information on the fluctuations in the horizontal wind components, from -10 m/s to 12 m/s and from -17 m/s to 14 m/s, and the vertical acceleration recorded by the QAR during this period also exhibits obvious fluctuations (0.98g-1.08g, 1.1g-0.96g), indicating that the multi-head attention mechanism can effectively fuse three-dimensional wind features to obtain wind data consistent with the trend of variation in aircraft turbulence.

**Fig 9:**
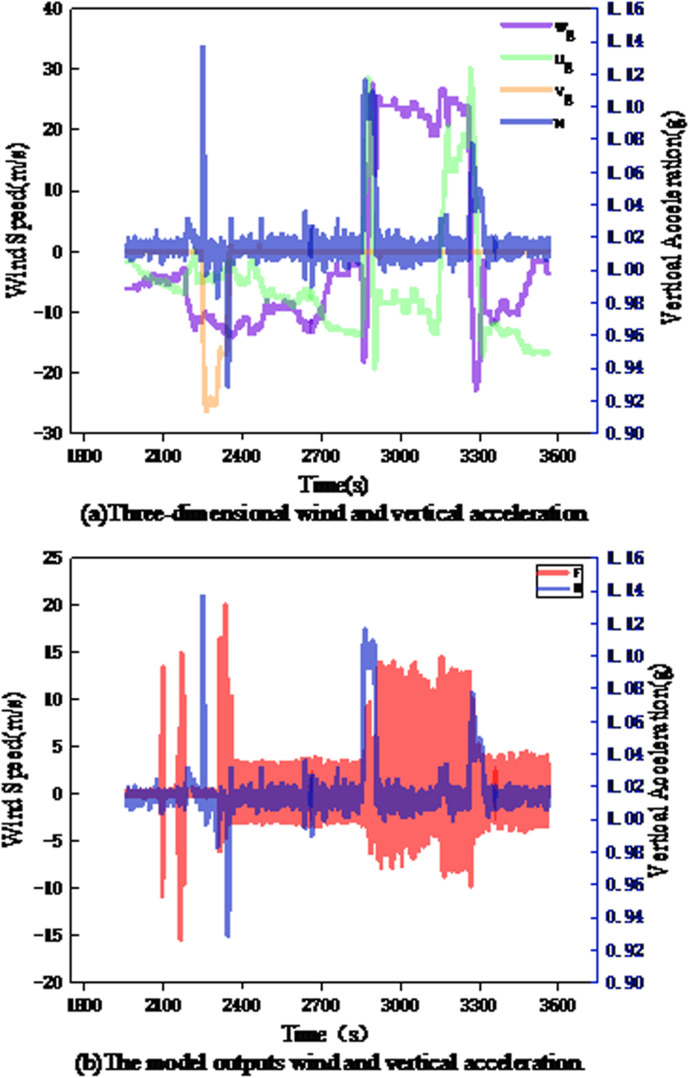
Wind and vertical acceleration of TF2 segment. (a)Three-dimensional wind and vertical acceleration; (b)The model outputs wind and vertical acceleration.

(2) TF2: On May 3,2022, a Boeing 737–800 aircraft flying from Chongqing Jiangbei International Airport to Jinan Yaoqiang International Airport (bumpy 50 minutes before descent in 2023) encountered mild turbulence 50 minutes before landing. The variation of wind and vertical acceleration during this period is shown in Fig 9.

Fig 9(a) shows the specific data of TF2.If it is divided by the time of 2600 s, it can be regarded as two mild turbulences in this section of the route: when the vertical acceleration changes for the first time, the fluctuation of horizontal wind (Ug and Vg) is small, which does not reflect the change of vertical acceleration, while the vertical wind (Wg) changes between -26 m/s and 1 m/s, and the violent fluctuation of vertical wind is the main reason for the turbulence of the aircraft at this time. After 2600 s, when the aircraft encountered the second turbulence, the change of vertical wind was relatively stable, and the fluctuation was basically 0, which did not reflect the change of vertical acceleration, while Ug and Vg changed between-24.3 m/ s and 29.6 m/ s. Obviously, the turbulence at this time was mainly caused by horizontal wind. As shown in Fig 9(b), the data obtained by feature fusion can not only effectively retain the fluctuation information of vertical wind (-26 m/ s to 1 m/ s), but also well reflect the fluctuation information of horizontal wind (-24.3m/ s to 29.6m/ s), which is consistent with the overall change trend of aircraft turbulence. This shows that the multi-head attention mechanism can effectively fuse the three-dimensional wind and supplement the aircraft oscillation caused by horizontal gusts.

### 5.4. Fusion effectiveness analysis

The sample dataset was used to train the proposed MHE, model for comparison with the VWE and PCE methods. The difference between PCE and MHE is that PCE uses PCA to fuse three-dimensional wind data. The three-dimensional wind data is are mapped to a new coordinate system via linear transformation, and the dimension with the largest variance is used as the result of feature fusion. The EDR data recorded as part of the QAR data are used to analyse the estimation performance of each method. The obtained scatter plots are shown below, where the reference line represents the specific grading relationship between the EDR and turbulence intensity as specified in Annex 3 of the ICAO International Convention [18].

**Fig 10:**
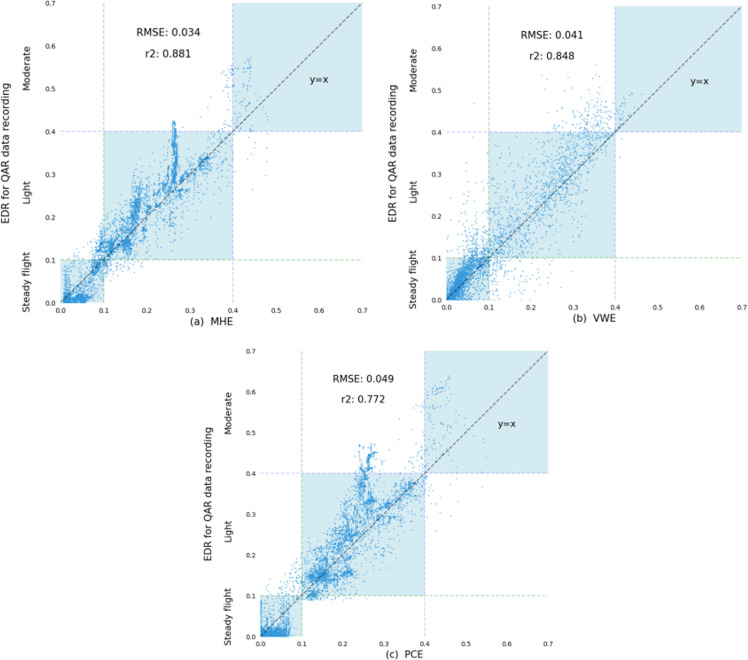
Comparison of EDR estimates. (a) Estimated performance metrics for MHE;(b) Estimated performance metrics for VWE;(c) Estimated performance metrics for PCE.

As shown in Fig 10, The scatter points represent the estimated EDR values. The R2 of MHE is 0.881, which is 3.3% and 10.9% greater than those of VWE and PCE, respectively. The root mean square error (RMSE) is 0.034, which is 0.7% and 1.5% lower than those of VWE and PCE, respectively. VWE considers only the fluctuations in the vertical wind and ignores the influence of the horizontal wind, which leads to underestimation of the EDR and possible, misjudgements of mild turbulence as no turbulence and moderate turbulence as mild turbulence.

On the premise of defaulting all three-dimensional wind components to the same weight, PCE selects the dimension with the largest variance in the coordinate system of the three-dimensional wind projection. However, this ignores the fact that aircraft are more sensitive to vertical wind, consequently exaggerating the influence of horizontal wind, and reducing the estimation accuracy. In contrast, MHE performs feature fusion by suitably weighting three-dimensional wind, data and relies on the split-head method to control the feature fusion weights more accurately, thus achieving superior overall estimation results.

## 6. Discussion and conclusions

The proposed algorithm provides highly accurate EDR estimates for flight routes, characterized by fast execution and low computational cost. It also demonstrates significant practical applicability in assessing turbulence intensity along these routes, thereby enhancing aircraft safety during flight.

This paper employs a multi-head attention mechanism to quantify the relationship between three-dimensional wind features and the feature matrix (vertical acceleration), correspondingly mapping the feature fusion weights resulting from this process onto the three-dimensional wind matrix. The three-dimensional wind data is then transformed into a one-dimensional form to achieve feature fusion, generating new wind data. This approach enhances the comprehensiveness of wind information, incorporating the new one-dimensional fused wind data in the frequency domain with maximum likelihood estimation can significantly improve the accuracy of EDR estimation. The experimental results indicate that the model achieved an R² (R-Square) of 0.881, representing improvements of 3.3% and 10.9% over EDR estimations based on Vertical Wind Estimation (VWE) and Principal Component Analysis Estimation (PCE), respectively. Moreover, the root mean square error (RMSE) is 0.034, which is 0.7% and 1.5% lower than those of VWE and PCE, respectively. The proposed algorithm can update the weights of three-dimensional wind characteristics in real time according to actual conditions, thereby incorporating horizontal wind fluctuations into the wind data used for EDR estimation while still emphasizing the impact of vertical wind, which effectively improves the accuracy of EDR estimation. Due to the limitations of the existing dataset, extreme cases have not been discussed; however, in future work, we plan to collect a feature dataset that includes extreme cases for further analysis.

**Highlights**

1. Fusion of the vertical wind and the two horizontal wind components data, providing more comprehensive turbulence information, Subsequently, by combining the new one-dimensional fused wind data in the frequency domain using maximum likelihood estimation, the accuracy of EDR estimation is significantly enhanced.
2. Using Spearman analysis, the correlation between the quality-controlled QAR data and EDR was analyzed, revealing that vertical acceleration had the strongest correlation with EDR. vertical acceleration is used to construct the feature matrix, then, using a multi-head attention mechanism to quantify the relationship with the three-dimensional wind characteristics. This process generates feature fusion weights that are mapped onto the three-dimensional wind matrix, enabling the conversion of the three-dimensional wind data into one-dimensional fused wind data.
3. The proposed algorithm provides high-accuracy EDR estimates for flight routes and demonstrates strong practical applicability in assessing turbulence intensity along the flight routes, thereby enhancing the safety of aircraft during flight.

## Supporting information

S1 Data (RAR)

## Abbreviations

EDR: Eddy Dissipation Rate
QAR: Quick Access Record
VWE: Vertical Wind Estimation
MHE: Multi-head attention mechanism Estimation
PCE: Principal Component Analysis Estimation
DEVG: Derived Equivalent Vertical Gust Velocity
MLE: Maximum Likelihood Estimation
